# The electronic NOSE and its application to the manufacture of food products

**DOI:** 10.1155/S1463924695000277

**Published:** 1995

**Authors:** Diana Hodgins, Derek Sirnmonds

**Affiliations:** 1 Neotronics Scientific Ltd Western House 2 Cambridge Road Stansted Mountfitchet, Essex CM24 8BZ UK; 2 Materials Research Institute Sheffeld Hallam University City Campus Sheffield S1 1WB UK

## Abstract

The Electronic NOSE (Neotronics Olfactory Sensing Equipment) is an instrument which mimics the human olfactory sensory system. It analyses complex vapours and produces a simple output. In the food industry there are numerous examples where the aroma from the raw ingredients through to the final product are important. These aromas are currently analysed using human sensory panels or analytical equipment such as gas chromatography/mass spectroscopy (GC/MS).

The Electronic NOSE described in this paper was not developed to replace the GC/MS or the sensory panel but to provide an instrumental measure of aroma quality which would be related to and complement the current methodology. The Electronic NOSE is a robust system which can detect complex vapours at levels similar to the human, which means typically in the parts per billion range. The system produces an output which can be easily related to sensory data and is easy to interpret by a non-skilled operator. No part of this system reacts with the sample under test.


Journal of Automatic Chemistry, Vol. 17, No. 5 (September–October 1995), pp. 179–185

### The electronic NOSE and its application to the manufacture of food products

**Diana Hodgins**
*Neotronics Scientific Ltd, Western House, 2 Cambridge Road, Stansted Mountfitchet, Essex CM24 8BZ, UK*

**and Derek Simmonds**
*Materials Research Institute, Sheffield Hallam University, City Campus, Sheffield S1 1WB, UK*

*The Electronic NOSE (Neotronics Olfactory Sensing Equipment) is an instrument which mimics the human olfactory sensory system. It analyses complex vapours and produces a simple output. In the food industry there are numerous examples where the aroma from the raw ingredients through to the final product are important. These aromas are currently analysed using human sensory panels or analytical equipment such as gas chromatography/mass spectroscopy (GC/MS).*

*The Electronic NOSE described in this paper was not developed to replace the GC/MS or the sensory panel but to provide an instrumental measure of aroma quality which would be related to and complement the current methodology. The Electronic NOSE is a robust system which can detect complex vapours at levels similar to the human, which means typically in the parts per billion range. The system produces an output which can be easily related to sensory data and is easy to interpret by a non-skilled operator. No part of this system reacts with the sample under test.*

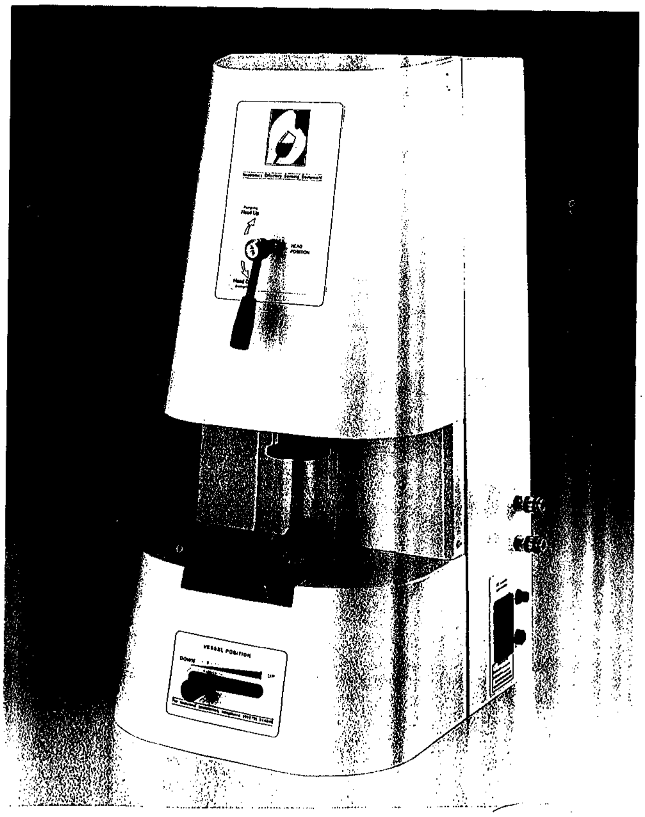

*Figure 2. Electronic NOSE.*

### Introduction

The NOSE (Neotronics Olfactory Sensing Equipment) which is described in this paper is shown in figures 1 and 2. The key aspects are the sensor array and the overall system design, both of which will be described in some detail.

The potential applications for the system within the food industry include all operations where the endpoint is defined by aroma. This covers on-line control for a range of processing including roasting, toasting, baking and cooking and blending operations for products such as sauces, soups and coffees. The NOSE could also be used for routine QA/QC operations such as new materials characterization in particular species, herbs and flavourings, authenticity monitoring and detection of off-flavours and taints.

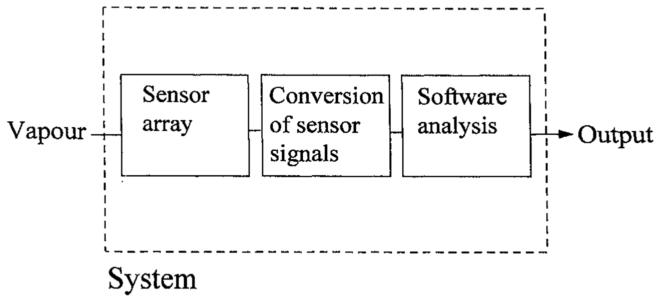

*Figure 1. Principal components of the NOSE.*

The flavour of foods is made up of two components: the taste which is caused by non-volatile compounds which react with biological sensors on the tongue and in the mouth; and the volatile compounds which interact with sensors in the nose and are responsible for odour. Of these, the many hundreds of volatiles which make up the odour are by far the most important in defining product type and individual preferences, and are responsible for off-flavours and taints which may arise in foods. Volatile odour compounds may arise in foods from a variety of sources—for example the process of toasting and roasting can generate a range of odorous compounds which import the typical roasted, toasted, nutty, odour associated with bread, breakfast cereals and snack foods. The types of compounds generated are formed via caramelization of sugars or through the Maillard reaction which generates such compounds as thiazoles, pyrazines and pyridines.

Off-odours and taints arise in foods due to decomposition caused by endogenous enzymes, contamination or chemical oxidation. There are many examples of these including

0142-0453/95 $10.00 © 1995 Taylor & Francis Ltd.

microbial spoilage which can lead to putrid, ammoniacal, musty/mouldy odours in a range of products due to sulphur volatiles, amines and chloroanisoles. Both enzyme and chemical oxidation can lead to a range of rancid off-odours, particularly in high lipid containing products due to the formation of aldehydes and ketones. Other taints which arise are to simple contamination, for example by spillable of oils, diesel or cleaning agents.

All of these compounds are typically present at low concentrations in the vapour phase and the human can detect these down to the parts per billion level or even lower. It is therefore necessary that any sensor used in an electronic system must respond to the different groups described and effectively be able to detect these at similar concentrations to the human.

### Conducting polymer sensors

The sensor materials used in the NOSE are electrochemically grown onto a silicon substrate. The resulting polymer, for example polypyrrole, is in an oxidized form containing cationic sites balanced by anions from the electrolyte and the composite is electrically conductive. Conduction occurs via electron transport and the charge carriers are associated with the cation sites of the polymer (probably polarons or bipolarons providing mobile holes for electron transport). The conductivity attained is sensitive to the presence or absence of substituents on the pyrrole rings and to their electron-donating or withdrawing character.

In addition to the polypyrrole chains, the conductive composite contains the counterions and at least some of the solvent used for electrode position. All three components contribute both to the inherent resistance and to the interaction of analyte species with the sensor. Some counterions and solvents also affect the stability of sensors, so that the 'sensor' is best thought of as the composite (rather than just the polypyrrole) and a good sensor is one that reproducibly recognizes analytes and has good stability. Sensing depends on a change in conductance of the conductor composite on interaction (which must be reversible) with the analyte. The change may result directly from interactions between the organic vapours and the polymer, or indirectly from interactions between the vapours and the solvent-counterion matrix, or both mechanisms may be operative. In any case the sensing activity will result from a change to the 'doping' of the polymer, i.e. the population of active charge carriers in its structure that contribute to the conductance.

Little has been published on the detailed microstructure of conductive polymer materials related to polypyrrole [1]; however, it is known that the counterions and the solvent produce consistent effects [2] and that the polarity of organic analytes contributes to changes in the conductance [3]. In addition, it is probable that the solubility of different analytes in the local phase of the sensor surface will also effect the conductance.

In order to increase understanding of the material and vapour interaction, Neotronics and Sheffield Hallam University are currently combining their theoretical knowledge of materials, including the structure formation down to molecular level. This work will enable future materials to be designed based on models developed for more specific applications and could make the existing device more diagnostic of structural type in some analytes.

One aspect worth noting is that the polymer material has a finite thickness: typically around 50 µm. The composition of the material is such that a large number of chains will form alongside one another and on top of one another. While the surface of the sensor is probably the primary region for the important interactions with analytes, it is likely that counterions and solvents are distributed between the polymer chains and throughout the bulk material.

It is clear that, in a three-dimensional structure, the problem of molecular interaction from an external vapour is far more complicated than for the simple two-dimensional structure already described. Fortunately, the complications of the three-dimensional form do not have to be fully analysed, since it can be assumed that the surface interactions are repeated within the material structure.

The sensors show good stability but, in the long term, there is a gradual change in conductance. While, initially, no *primary* bonding occurs between the polypyrrole chains and the counterions, solvent or the analyte, it seems likely that small molecules become lodged in interstitial spaces, probably leading to local distortions of the conductive structure and hence a slight reduction in conductivity. Polar, donor molecules such as water (that can associate with cation sites) are probably responsible and observations confirm that prolonged exposure to high humidity levels is deleterious. The result is that the conductance of the polymer will reduce with time due to fewer charge carriers being present. However, the charge carriers that remain will not have been noticeably affected. Therefore, since the system monitors the percentage change in conductance or exposure to an organic vapour, the sensitivity will remain relatively constant.

### Sensors

Sensor technologies which are either available in production or as prototypes include tin oxide sensors [4]; conducting polymer sensors [5–7]; surface acoustic wave devices [8]; and biosensors/enzyme sensors [9]. Each of these offers different characteristics in terms of their selectivity and sensitivity to different compounds. Conducting polymer sensors offer the largest range in terms of selectivity and sensitivity and therefore this sensor technology has been adopted for the NOSE.

### *Conducting polymer sensors*

The sensor materials are electrochemically grown onto a silicon chip. The sensors are unique: first, the materials, which comprise a conducting polymer, a counterion and a solvent, are grown from solution onto a working electrode which is separated by a 10 µm gap. As the material grows it bridges the gap, thus producing a simple resistor. Each sensor type has either a different polymer, counterion or solvent, which alters its response to complex

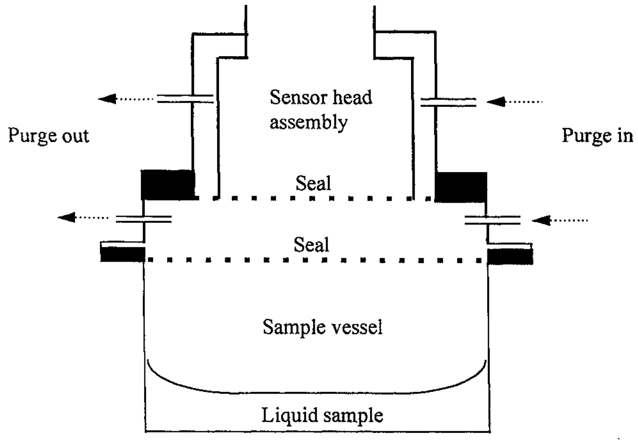

*Figure 3. Schematic of purging method.*

vapours. This very simple, single stage fabrication process produces the same matrix structure every time and therefore sensor to sensor reproducibility is extremely good. Second, semiconductor technology is used to produce a gas sensor; and 10 µm gaps are easy to reproduce consistently.

*Sensor array design*

To achieve a fingerprint from a complex vapour which may contain many thousands of molecules, it is necessary to have an array of sensors. A sufficient number of sensors is required in the array to ensure that a unique fingerprint is obtained from different samples. The array described has 12 sensors which are soldered onto a printed circuit board, which is then mounted into a sealed enclosure. Each sensor in the array uses a different polymer structure, and this structure defines the response characteristic of the sensor to complex vapours. It is very difficult to state which polymer material responds to individual compounds, since these generally exist in a complex form in the vapour. However, sensors can be chosen by utilizing the sensor response characteristics to known individual compounds. When testing complex vapours, a unique fingerprint will be obtained. The user can relate each fingerprint to sensory panels and/or GM/MS data if desired. Therefore for different applications the user will have a library of fingerprints which can be matched to different sensory attributes or flavour chemicals.

### System design

The system has been designed to ensure that optimum performance is obtained and that it is suitable for use in those industries for which it is designed. In the food industry, sensory panels place the samples in inert glass vessels, allow the sample to equilibrate into the headspace and then smell the headspace. The system has been designed to simulate this methodology as closely as possible. The sample container is glass and the sample is left to equilibrate before the sensors are exposed to the static headspace above the sample. All other materials that come into contact with the sample or the vapour are manufactured from inert materials to ensure that there is no contamination of the sample, nor any carry-over from one sample to another.

A second important consideration is that the samples must not be affected by environmental changes. To prevent this, the sample vessel and sensor array enclosure are purged with a known reference gas, which is typically dry air. Thus the sample equilibrates into the known reference gas and this is also the reference for the sensors. A schematic of the purging system is shown in figure 3.

The system is designed to work at room temperature, which closely correlates with sensory panel data. However if the sample changes with temperature and this variation is important then the system can be trained to recognize the sample at different temperatures.

### Sample testing

The system requires minimal sample preparation, with samples being presented to the NOSE whole, ground or pureed. In most applications the sample is placed into the sample vessel and allowed to equilibrate. The sensor array is then exposed to the headspace above the sample. The sensors respond to the vapour in a transient nature, with 90% of the response achieved within 30 to 60 seconds, depending upon the sample type. The test period is set for typically 60 s and the data is stored from all sensors every second during this period. On completion of the test, the sensor array is resealed from the sample vessel. The system has also been designed to allow for multiple sampling.

### Operation

Operation is simple, the operator is initially requested to enter information regarding the sample to be tested. This will include:

(1) *Description* of the test, for example a test identification number.

(2) *Class* which specifies the product group.

(3) *Substance* which specifies the member of a class.

(4) *Taints* which specifies any known impurities in the sample.

(5) *Effects* which describes the result of a taint or number of taints.

(6) *Comments* for any other information the operator wishes to record alongside the test data.

(7) *Method* which defines the sensors to be used and the duration of the acquisition for the specified class.

Once the details have been entered, acquisition can commence and the operator is kept informed of progress via a real time graphical profile. When acquisition is complete, the graphical profile of sensor response against

Sample

| | |
|---|---|
| Class | sesame oil |
| Substance | sample 3- desired taste |
| Sample | Average(Template) |
| Method | N/A |
| Time | N/A |
| Calibrated | None |

Cursor at 01:00

| | | | | | | | |
|---|---|---|---|---|---|---|---|
| +0.28 | Type 278 | +0.37 | Type 264 | +0.25 | Type 263 | -0.03 | Type 262 |
| +0.13 | Type 261 | | Type 261 | +0.20 | Type 260 | | Type 260 |
| +0.54 | Type 259 | +0.27 | Type 258 | +0.38 | Type 257 | | |

Sample Comments :
N/A

*Figure 4. Offset polar of fresh oil.*

time is automatically sealed. The data is then ready for further analysis.

### Analysis techniques

A number of analysis techniques could be used for the application, for example graphical representation of the individual sensor outputs with time; polar plots or spider plots; statistical techniques; offset polar or difference plots; neural networks.

For simple QC/QA type testing the last two examples would be suitable.

### Comparison of samples to a known reference

In the majority of cases users will want to monitor a product and determine whether it has deviated significantly from the reference sample. Using the difference plot technique any sample can be quickly compared to a known reference. If both sample and reference were identical then a circle would be drawn. If the sample output is less than the reference then the vector moves inwards and if it is greater then the vector moves outwards. Again the radius defines the magnitude of this difference. An example is the testing of two edible oil samples. The first was passed by a sensory panel, while the second was

**Analysis : Sample Average - Offset Polar view**

| | Sample |
|---|---|
| Class | sesame oil |
| Substance | sample 4 - bitter taste |
| Sample | Average(Template) |
| Method | N/A |
| Time | N/A |
| Calibrated | None |

Cursor at 01:00

| | | | | | | | |
|---|---|---|---|---|---|---|---|
| +0.22 | Type 278 | +0.26 | Type 264 | +0.20 | Type 263 | 0.00 | Type 262 |
| +0.12 | Type 261 | | Type 261 | +0.18 | Type 260 | | Type 260 |
| +0.49 | Type 259 | +0.35 | Type 258 | +0.36 | Type 257 | | |

Sample Comments :
N/A

*Figure 5. Offset polar of rancid oil.*

stated as rancid. The two individual plots are shown in figures 4 and 5 and the difference between the two shown in figure 6. It is important to note that the offset polar plots for the fresh and rancid oil samples are plotted on a 1.00% radius plot and the difference plot on a more sensitive scale with a radius of 0.20%. It is clear from the difference plot that there is a significant difference between the two samples shown on at least 50% of the sensors. In other examples a difference may only be seen on two sensors, which would suggest that a taint was present; if all sensors exhibited a difference then the sample would be said to be very noticeably different.

### Neural networks

The analysis techniques described so far are suitable for comparing samples to a specified reference. This is adequate when there is only one reference but becomes more complicated when there are a number of references.

Analysis : Difference (S(Avg)-R(Avg)) - Offset Polar view

| | Sample | Reference |
|---|---|---|
| Class | sesame oil | sesame oil |
| Substance | sample 4 - bitter taste | sample 3- desired taste |
| Sample | Average(Template) | Average(Template) |
| Method | N/A | N/A |
| Time | N/A | N/A |
| Calibrated | None | None |

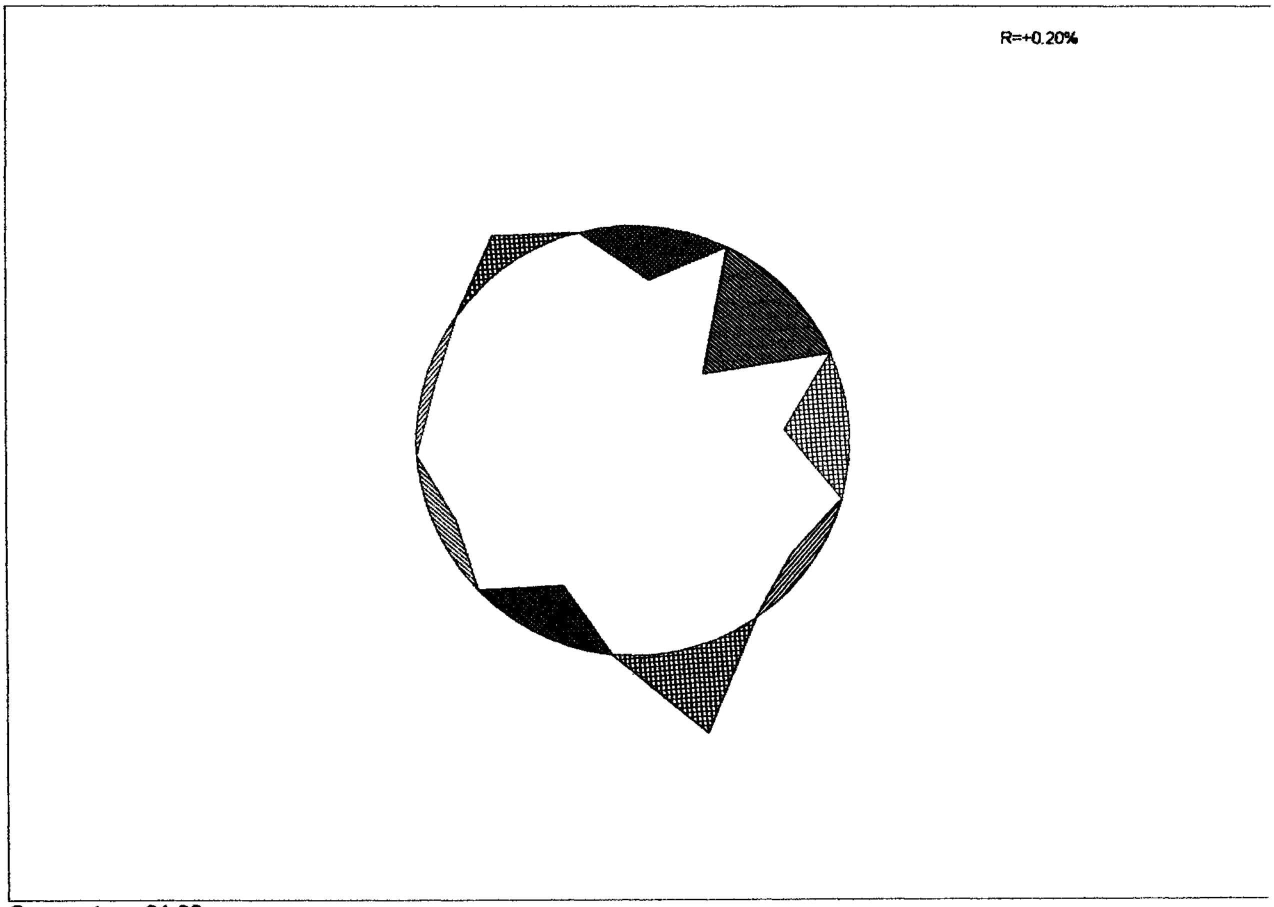

Cursor at 01:00

| | | | | | | | |
|---|---|---|---|---|---|---|---|
| -0.06 | Type 278 | -0.11 | Type 264 | -0.05 | Type 263 | +0.03 | Type 262 |
| -0.01 | Type 261 | | Type 261 | -0.02 | Type 260 | | Type 260 |
| -0.05 | Type 259 | +0.08 | Type 258 | -0.02 | Type 257 | | |

Sample Comments :
N/A

Reference Comments :
N/A

*Figure 6. Difference plot between the two oils.*

For example, if a user wishes to compare a sample whisky to six different whisky types using the techniques so far described, the user would have to compare the sample to one reference at a time, determining any differences. This would be quite a time-consuming process as the number of references increased.

A solution would be to use a neural network to analyse the data from the sensor array. In the simplest arrangement the percentage change in resistance at specified times for each of the sensors would be used as the inputs to the neutral network. The outputs, defined by the user, would be different known types, for example all the different whisky types.

There are a number of different neural networks that could be used for this task. One of the simplest has been successfully used with data obtained from both an eight and a 12 sensor array. In this case there were either eight or 12 sensor inputs and four to six outputs with one hidden layer. The artificial neurons carry out a simple summation using the weighting factors from all the links connected to the neuron. An arbitrary set of weights is given to the neural network before it is trained.

### Applications

The NOSE has been applied to both fresh and processed food, for example to test the *freshness* of fruit when the product is picked, during shipment and at the point of sale; the *maturing process* of cheese and product characterization; *quality assurance* checks on high value flavouring materials; and the identification of different *cuts of meat* to reduce potential packaging problems.

The NOSE has also been applied to both alcoholic and non-alcoholic beverages, for example in the identification of coffee bean types; instant coffee aromas during processing; the detection of diacetyl, dimethyl sulphide and amylacetates during fermentation; and the detection of trichloroanisole in both corks and packaging.

Further industries where the NOSE has been applied include tobacco; flavours and fragrances; packaging; petrochemicals; pharmaceuticals; water; power; and health care.

### References

1. Chandler, G. K. and Pletcher, D., *Electrochemistry*, R.S.C. Specialist Periodical Reports, **10** (1985), Chapter 3.
2. Zhang, W. and Dong, S., *Electrochimica Acta*, **38** (1993), 441.
3. Kunugi, Y., Nigorikawa, K., Harima, Y. and Yamashita, K., *Journal of the Chemical Society*, **863** (1994).
4. Shurmer, H. V., Gardner, J. W. and Chan, H. T., *Sensors and Actuators* (1989).
5. Bartlett, P. N., Archer, P. B. M. and Ling-Chung, S. K., *Sensors and Actuators* (1989).
6. Bartlett, P. N. and Ling-Chung, S. J., *Sensors and Actuators* (1989).
7. Diaz, A. F., Jkanasawa, K. K. and Gardin, G. P., *Journal of the Chemical Society*, **635** (1979).
8. Wohltzen, H., *Sensors and Actuators* (1984).
9. Higson, S. P. J., Reddy, S. M. and Vadgama, P. M., *Engineering Science and Education Journal* (February 1994).

